# Phytochemical and biological review of *Aegle marmelos* Linn

**DOI:** 10.2144/fsoa-2022-0068

**Published:** 2023-03-23

**Authors:** S Monika, M Thirumal, PR Kumar

**Affiliations:** 1 Department of Pharmacognosy, SRM College of Pharmacy, SRM Institute of Science & Technology, Kattankulathur, Chengalpet Dt, Tamilnadu, 603203, India

**Keywords:** *Aegle marmelos*, Marmelosin, phytochemistry, Rutaceae, Vilvam

## Abstract

India has one of the most expanded plant-origin medical traditions in the world. Researchers have evaluated molecules obtained from plants to treat a variety of ailments. Literature review shows that fundamental parts of the plant are used to treat different diseases. The related data is retrieved from Google scholar, PubMed, Science Direct and Scopus. The keywords include Bael, *A. marmelos*, Vilvam, and Marmelosin. Extensive studies show that *A. marmelos* has antidiarrhoeal, antimicrobial, antiviral, anticancer, chemopreventive, antipyretic, ulcer healing, antigenotoxic, diuretic, antifertility, and anti-inflammatory properties. In this work, an updated literature review is presented to clarify the current state of research on *A. marmelos* elucidating its constituents and their most relevant biological activities.

## Plain language summary

India has one of the most expanded plant-origin medical traditions in the world. *A. marmelos* Linn, also familiar as bael, belongs to Rutaceae and is widely grown worldwide. *A. marmelos* is a fruit with various medicinal advantages. We searched various databases, studied elaborately, and understood the importance of this fruit. Thus, its constituents can help mitigate various diseases.

## Graphical abstract

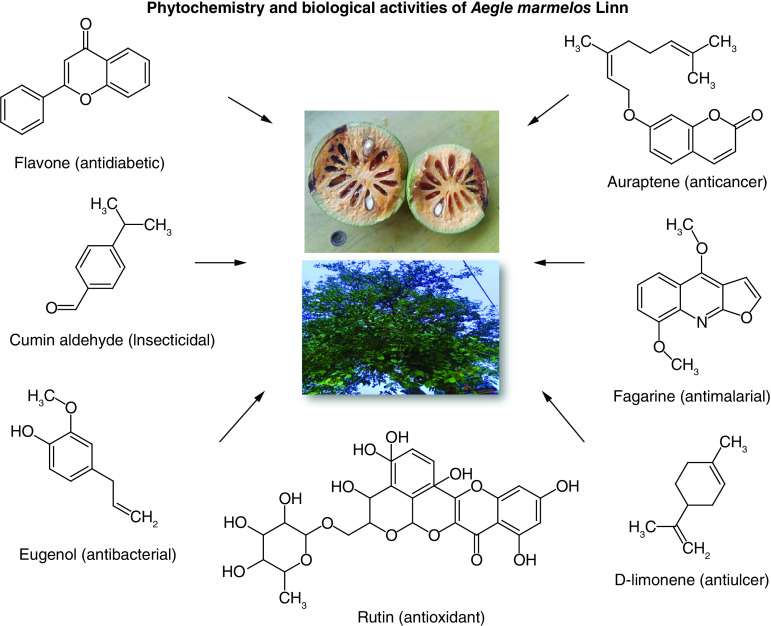

The medicinal plant performs an essential role in the lives of underprivileged populations worldwide [1], likewise for primary medical care. Approximately 80 percent of countries worldwide rely on these conventional treatments, which frequently involve plant extracts [2]. India has one of the most expanded plant-origin medical traditions in the world. In India, rural communities know around 25,000 potent plant-based remedies employed in traditional medicine. Plants, especially those with ethno pharmacological uses, have been the primary sources of medicine for early drug discovery [3]. Anciently most medications have been developed via natural ingredients or ingredients derived from natural compounds [4,5].

However, a significant amount of basic and applied research is required to validate and use plants in phytopharmaceutical chemistry, and the potential use of higher plants as a source of new medications is still underutilized, with this resource ranking on par with conventional pharmaceutical products in terms of importance [6]. Only a small portion of the approximated 250,000–500,000 plant genera have been thoroughly explored in terms of their pharmacological qualities, and only a small portion have been investigated phytochemically [7]. By supporting the conscious exploration of biodiversity as a source of bioactive molecules and their application in the production of new therapeutic medications, it also aims to encourage the developing and disseminating of this plant-based medicine. The main aim of this review is to know the phytochemical parameters, Traditional uses, and innovative applications of *A. marmelos* Linn.

## Research method

The search is done in Google scholar, PubMed, Science Direct and Web of Science. The databases are collected by the following keywords: Bael, *A. marmelos*, Vilvam, and Marmelosin.

## Inclusion & exclusion criteria

The language of this study is English. It included chemical, and pharmacological data and specific animal trials using isolated chemicals and extracts from *A. marmelos*. Finally, to ensure dependability, only peer-reviewed academic publications are selected. This study excluded the clinical trials and computational and characterization studies. 350 articles were selected; from that, 79 articles were included.

## Rutaceae family

The most recent phylogeny for Rutaceae, with 135 genera representing 87.7% of the recognized genera for Rutaceae and subfamilies of the family are Haplophylloideae, Amyridoideae, Aurantioideae, Cneoroideae, Rutoideae, and Zanthoxyloideae. The physiologically active essential oils produced by the Rutaceae family are widely known and found in many family members, as well as its ornamental and culinary herbs, which include orange, lime, lemon, grapes, and satinwood [8]. Several studies have found various plant substances, including alkaloids, terpenoids, flavonoids and coumarins [9]. Plants in the Rutaceae family contain high amounts of coumarins, like Marmelosin and Luvangetin, which have antihelminthic, antiulcer, antibacterial and antispasmodic activity.

### *Aegle marmelos* Linn

**Figure 1.**
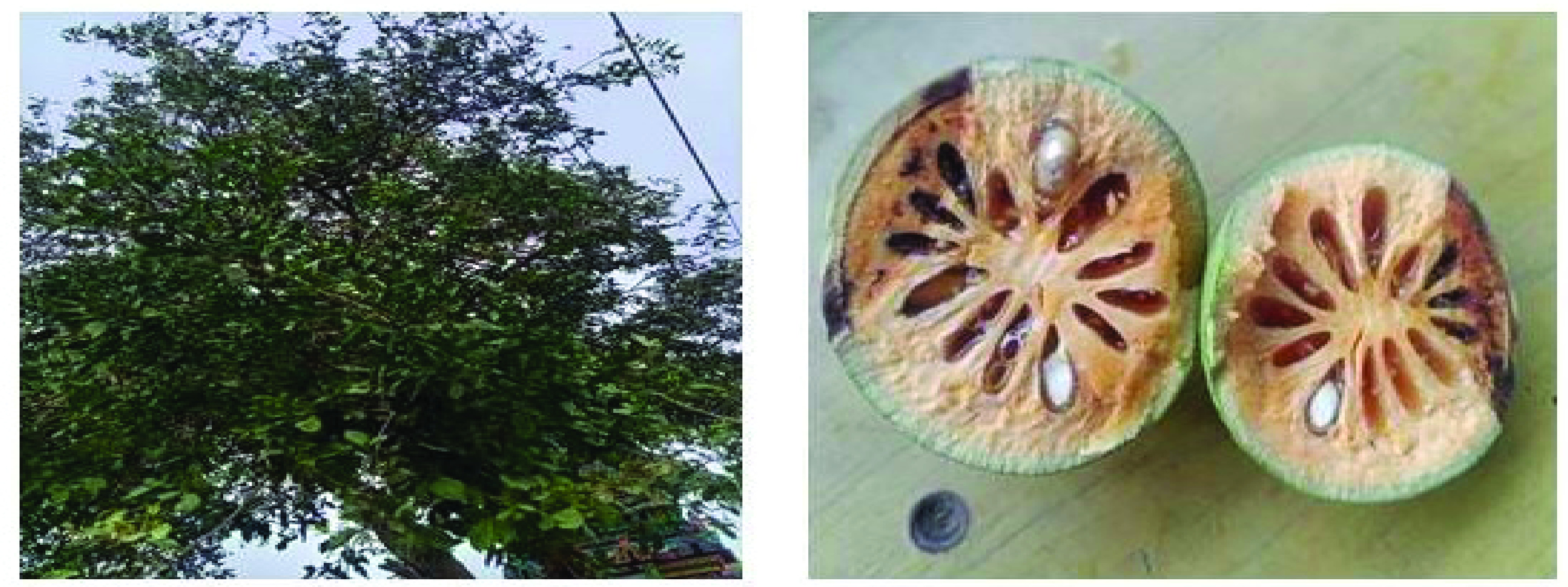
Bael fruit and tree.

*Aegle marmelos* Linn, also familiar as Bael as shown in Figure 1 and belonging to the family Rutaceae, has been frequently utilized in the indigenous Indian system of medicine because of its diverse medicinal properties. India holds high regard for the critical medicinal herb *A. marmelos* Linn (Rutaceae), also called Bengal quince, Bilva, Indian quince, Golden apple, Holy fruit, Bel, Belwa, Sriphal, Stone apple, and Maredo in India [10]. It has been utilized for over 5000 years by numerous ethnic populations living in the Indian subcontinent. In the ayurveda Indian traditional medicine system, it is used to treat various ailments [11]. The phytochemicals of *A. marmelos* were discovered in various sections of the same plant.

Indian medicinal plant known as bael has been used traditionally to treat several ailments, and numerous bioactive chemicals have been extracted [12,13]. *A. marmelos*, native to Northern India, are also widely dispersed over the Indian Peninsula, Burma, Bangladesh, Ceylon, Thailand and Indo–China [14]. The medium-sized, slow-growing *A. marmelos* tree can grow to 12–15 meters. It spreads with spiky branches and has a small trunk and thick, soft, peeling bark. Fractured branches, a transparent, viscous liquid that resembles gum arabic, oozes out, hangs down in long strands, and gradually solidifies. It starts tasting sweet but soon becomes unpleasant to the throat [15,16].

The biologically active chemicals and essential oil were extracted from Bael plants, and phytoconstituents characterization was carried out. Extraction techniques are used on the most active parts of the plant (roots, fruit, leaves, flowers, or stem), using selective solvents and standard operating procedures [17]. The taxonomical classification of *A. marmelos* is discussed in Table 1 [18].

**Table 1.** Taxonomical classification .

Kingdom	Plantae
Sub-kingdom	Tracheobionta
Super division	Spermatophyta
Division	Magnoliophyta
Class	Magnoliopsida
Subclass	Rosidae
Order	Sapindales
Family	Rutaceae
Genus	*Aegle*
Species	*marmelos*

In traditional medicine, *A. marmelos* are used based on their radio protective [19], antidiabetic, and anticancer activities [20,21]. The various components of bael are used for its medicinal properties, such as managing asthma, fractures, anemia, wound healing, high blood pressure, jaundice, swollen joints, diarrhoea, and issues with typhoid during pregnancy [22]. The medicinal importance of *A. marmelos* has been discussed in Table 2 [23–28] focusing on each part of the plant.

**Table 2.** Ethno medicinal uses of *Aegle marmelos* .

Parts	Uses
Leaves	The leaves are most effective in treating fever, nausea, vomiting, swellings, dysentery, dyspepsia, seminal weakness, and intermittent fever.
Root	The roots of bael are thought to be effective in treating urinary problems, preventing heart palpitations, and curing fevers. They are also said to relieve abdominal pain. The medical properties of dashamula lie in its root to treat fever, diarrhea, and flatulence.
Bark	The villagers use a decoction of the bark to treat fever and cough.
Flower	An anti-dysenteric, antidiabetic, diaphorectic, and local anesthetic medication can be produced by distilling flowers. It is utilized as a tonic for the stomach and intestine. Along with being used as an expectorant, it is also helpful in epilepsy.
Fruit	Bael fruits are edible. The pulp used to make delicious items like murabba, puddings, and juice. Apart from their laxative use and curing respiratory ailments, also used in several traditional medications to treat chronic diarrhea, peptic ulcers, inhibit lipid peroxidation, free radicals scavenging, antioxidants, anti-ulcerative colitis, gastroprotective, hepatoprotective, antidiabetic, cardioprotective, radioprotective, antibacterial, antidiarrheal and antiviral properties.
Seed	Seed extract possesses antidiabetic and hypolipidemic effects in diabetic rats.

*A. marmelos* is reported to contain chemical composition like alkaloids (aegeline, fragrine, aegelenine), coumarins (Marmin, Marmelide, Psoralen, Imperatonin), and terpenoids (cineol, Caryophyllene), etc [29–36].

## Reported phytochemical & its activity

The pulp of the bael fruit is rich in bioactive substances such as carotenoids, phenolics, alkaloids, pectins, tannins, coumarins, flavonoids, and terpenoids, according to studies. Methanol and water are the best solvents for extracting the metabolites of this plant, followed by ethanol [37–40]. The phytochemistry of *A. marmelos* has been extensively studied, and the plant has been found to contain a variety of biologically active compounds.

Some of the key phytochemicals found in *A. marmelos* include: Alkaloids are nitrogen-containing compounds that are found in many plants and are known for their pharmacological activity. Several alkaloids have been identified in the leaves and roots of *A. marmelos*, including marmesin, marmelosin, and aegeline [25].

Tannins are a group of compounds that are widely distributed in the plant kingdom and are known for their astringent and antioxidant properties. The fruit of *A. marmelos* contains high levels of tannins, which have been shown to have strong antioxidant and anti-inflammatory activities. Flavonoids are a group of compounds that are widely distributed in the plant kingdom and are known for their anti-inflammatory, anti-cancer, and antioxidant activities.

Flavonoids have been identified in the leaves and roots of *A. marmelos*, and some of these compounds have been shown to have antinociceptive (pain-relieving) and antipyretic (fever-reducing) activities [41].

Terpenoids are a group of compounds that are widely distributed in the plant kingdom and are known for their medicinal properties. Terpenoids have been identified in *A. marmelos*, and some of these compounds have been shown to have antifungal and antibacterial activities.

Saponins are a group of compounds that are widely distributed in the plant kingdom and are known for their foaming and emulsifying properties. Saponins have been identified in the fruit and leaves of *A. marmelos*, and some of these compounds have been shown to have antinociceptive and anti-inflammatory activities [33].

Glycosides are a group of compounds that are widely distributed in the plant kingdom and are known for their medicinal properties. Glycosides have been identified in the fruit and leaves of *A. marmelos*, and some of these compounds have been shown to have antinociceptive and anti-inflammatory activities [42]. The most widely investigated compounds from *A. marmelos* were determined by reviewing and evaluating the items from the obtained bibliographic data. The isolated phytochemicals from different parts of *A. marmelos* are discussed in Table 3, and the chemical structure of the compounds is shown in Table 4 [43].

**Table 3.** Compound isolated from various parts of *Aegle marmelos*.

S. no	Parts	Chemical compound	Ref.
i)	Leaves	α and β sitosterol, Rutin, Flavone, Cineol Glycoside, O- Halfordiol, Marmeline, Lupeol, Citronellal, Marmesinin, Aeglin, Cuminaldehyde, Phenylethyl cinnamamides, Citral, Skimmianine, Eugenol, Isopentenyl.	[44]
ii)	Fruit	Aurapten, Imperatorin, Psoralen, Tannin, Luvangetin	[45]
iii)	Bark	Fagarine, Marmin	[37]
iv)	Seed	Citral, A-D-phellandrene, Cineol, P-cymene, D-limonene, Cumin aldehyde, Citronellal	[46]

**Table 4.** Chemical structures of compounds present in *Aegle marmelos*.

S. no	Compounds	Molecular formula	Molecular Structure	Activity
1.	Aegelin	C_25_H_22_O_11_	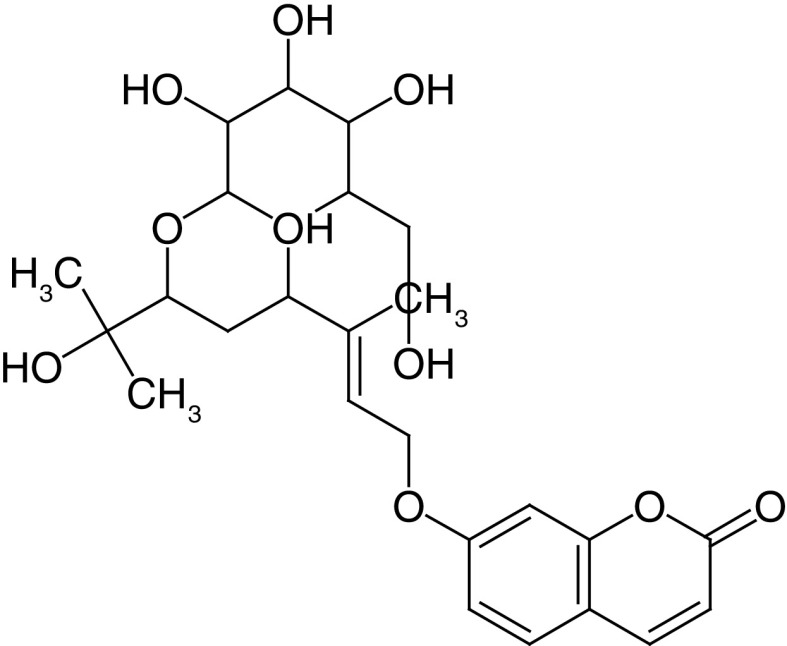	Antidiabetic
2.	Auraptene	C_19_H_22_O_3_	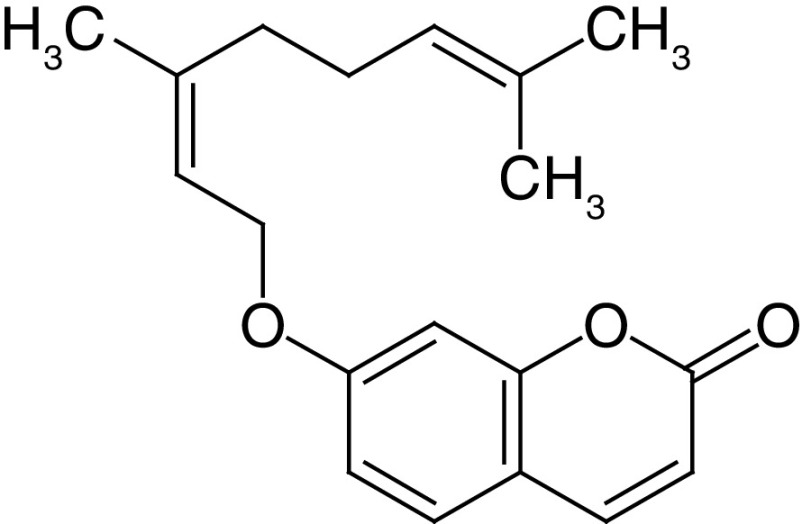	Inhibition of heart rate
3.	Cineol	C_10_H_18_O	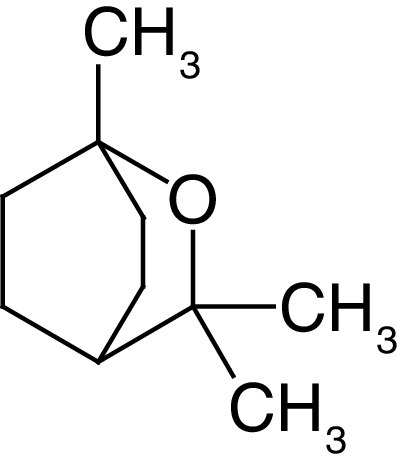	Expectorant, Disinfectant
4.	Citral	C_10_H_16_O	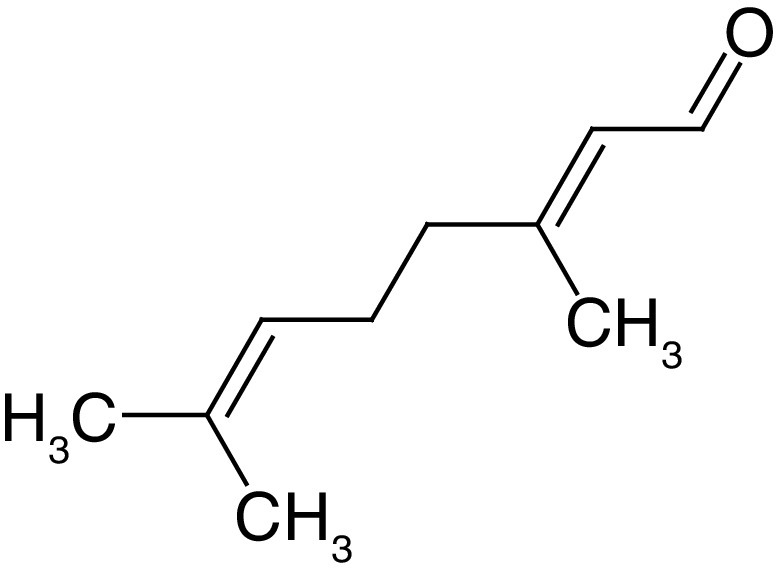	antibacterial, antifungal, and antiparasitic
5.	Citronellal	C_10_H_18_O	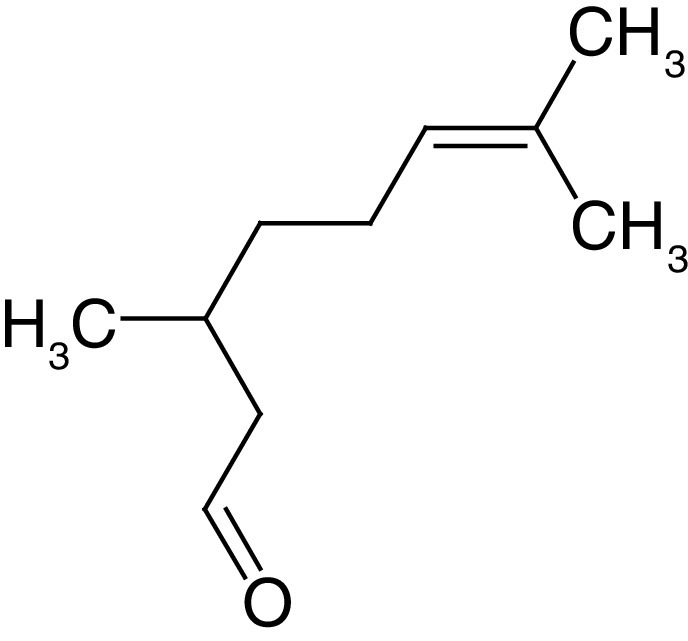	Anticancer, antiseptic
6.	Cumin aldehyde	C_10_H_12_O	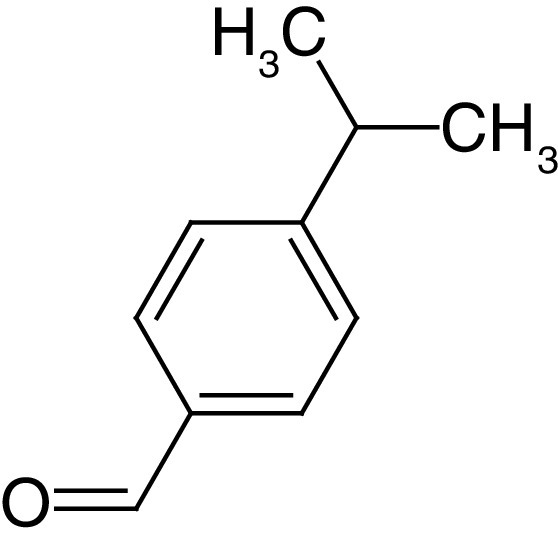	Insecticide
7.	D-limonene	C_10_H_16_	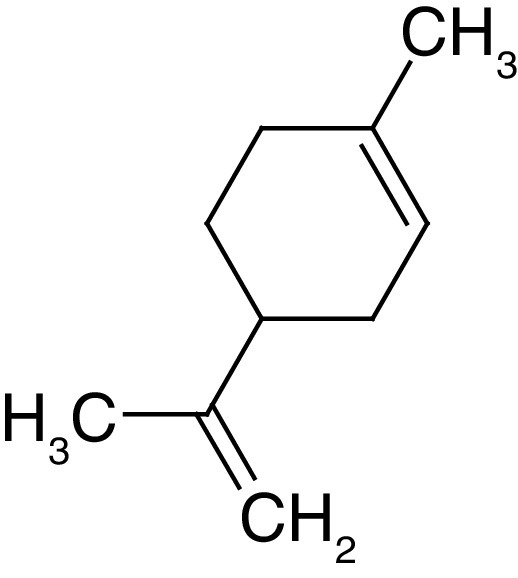	Dissolve cholesterol-containing gallstones
8.	Eugenol	C_10_H_12_O_2_	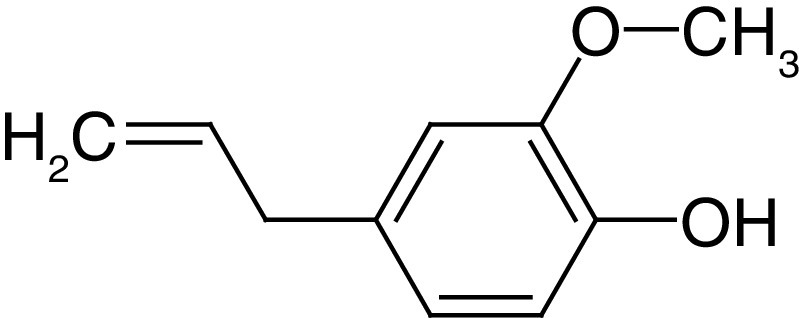	antibacterial, analgesic, and antioxidant
9.	Fagarine	C_13_ H_11_NO_3_	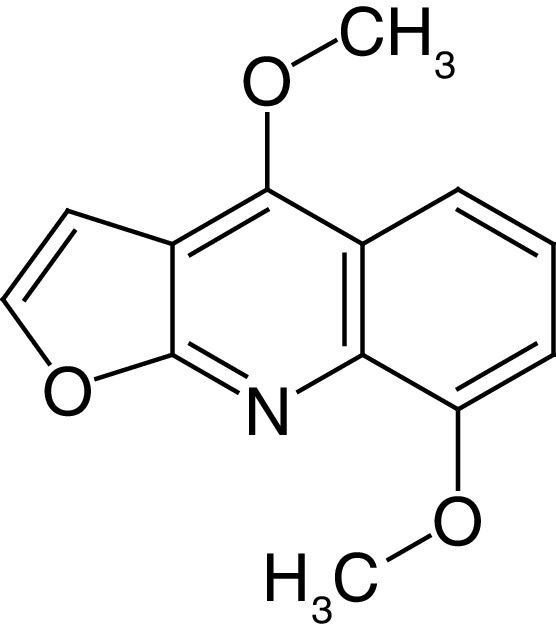	Antiplasmoidal
10.	Flavone	C_15_H_10_O_2_	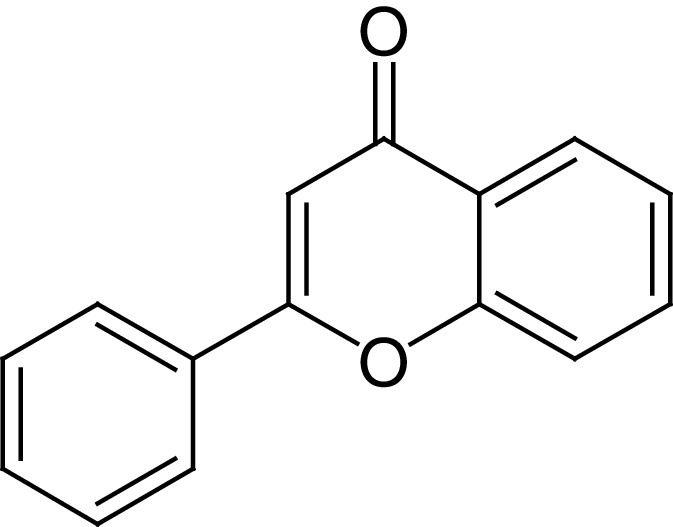	antifungal
11	Imperatorin	C_16_H_14_O_4_	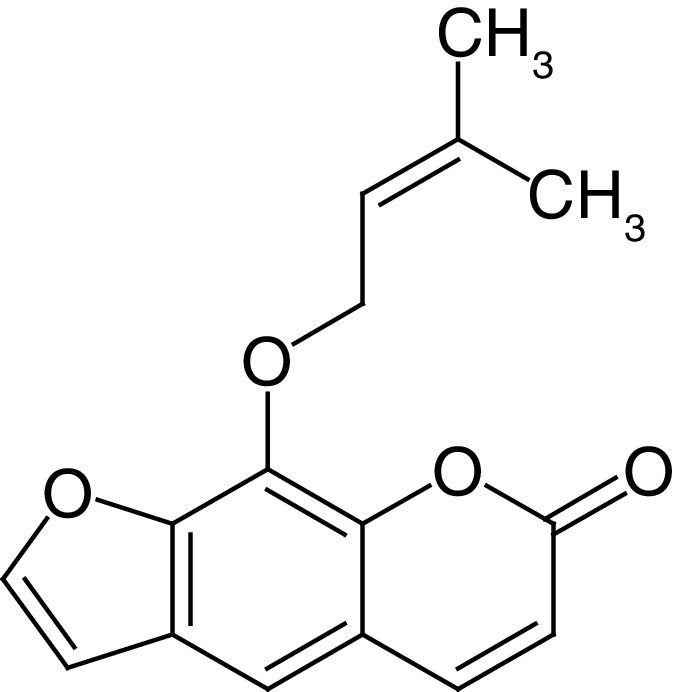	Antiviral
12.	Luvangetin	C_15_H_14_O_4_	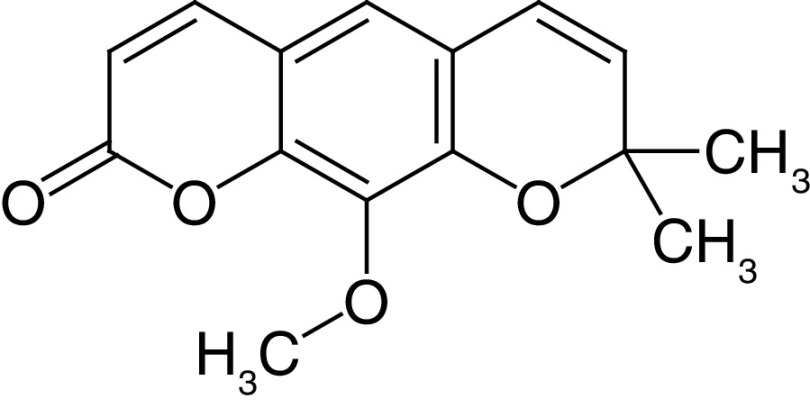	Antiulcer
13.	Marmin	C _19_H_24_O_5_	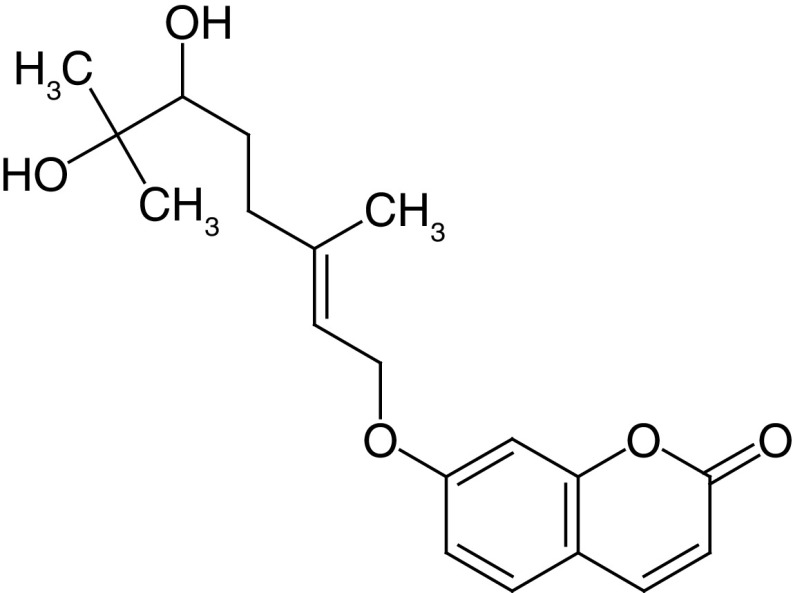	Antiulcer
14.	P-cymene	C_12_H_14_	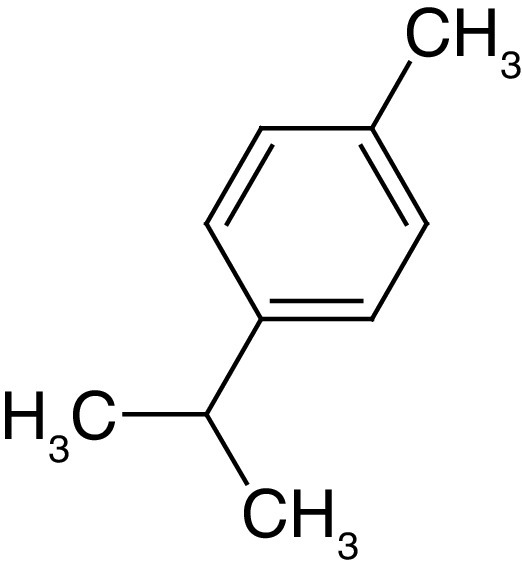	antiviral, antitumor, antibacterial, and antifungal.
15.	Psoralen	C_22_H_6_O_3_	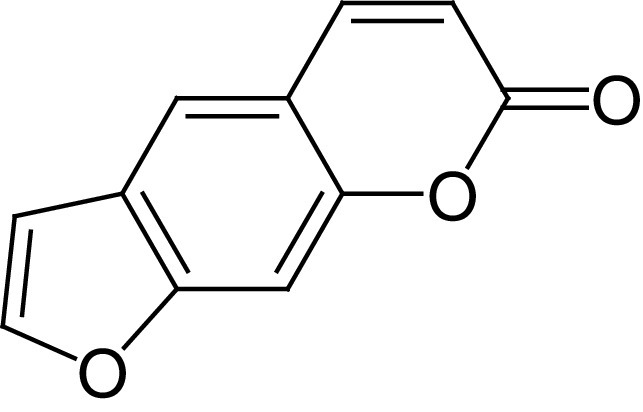	Cytotoxic, antispasmodic
16.	Rutin	C_2_H_30_O_16_	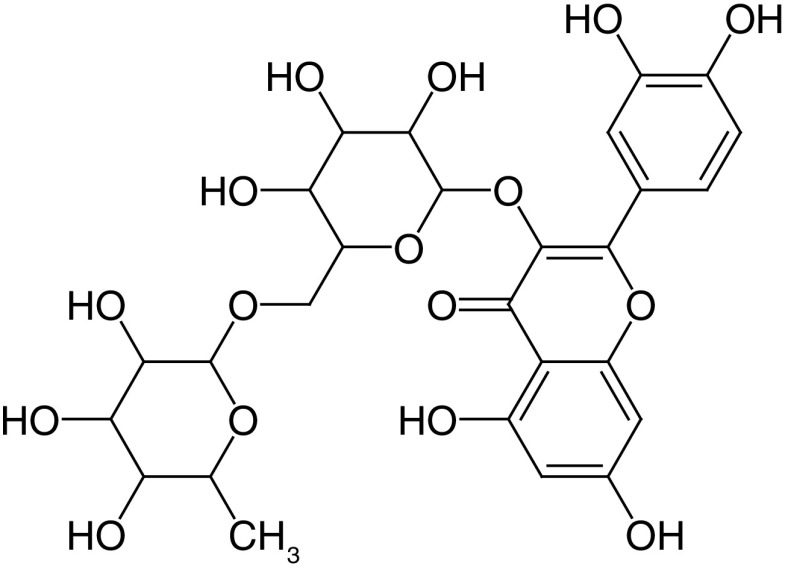	Antioxidant, Anti-inflammatory
17.	Skimmianine	C_13_H_14_NO_4_	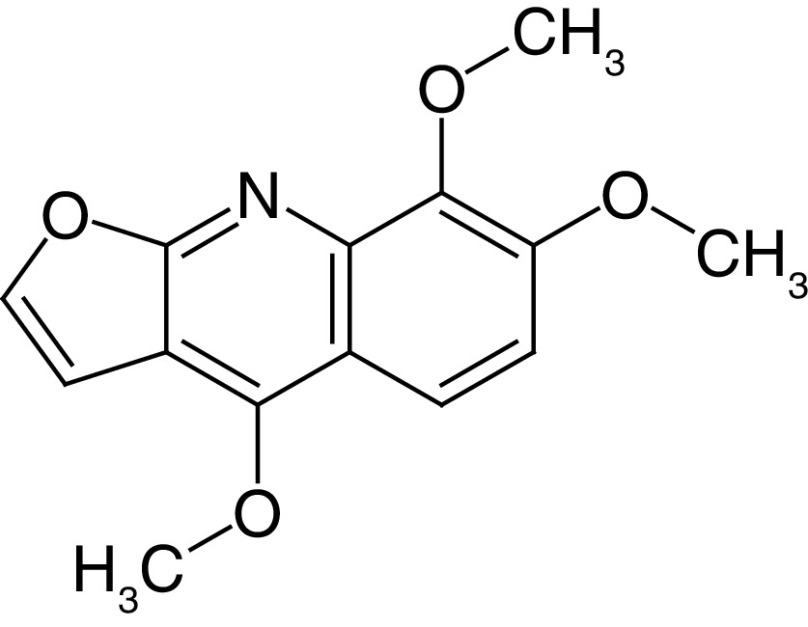	Sedative, anticonvulsive, analgesic
18.	β sitosterol	C_29_H_50_O	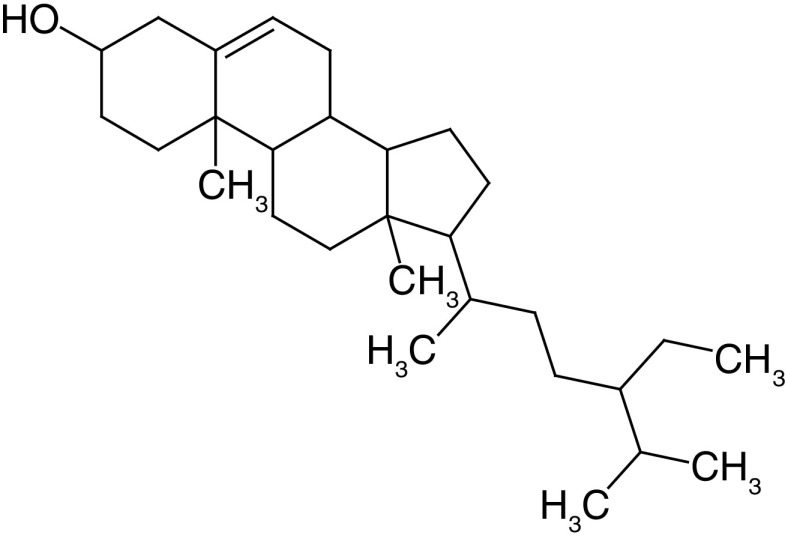	Antioxidant

## Pharmacological activity

**Figure 2.**
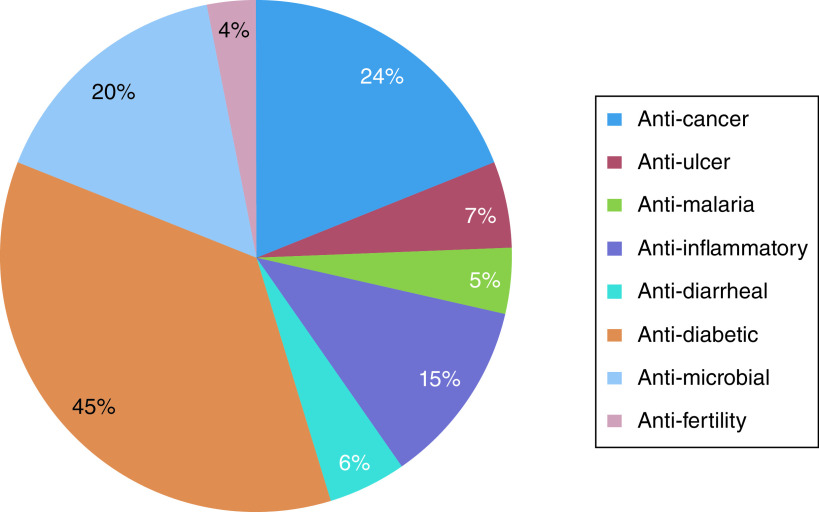
Percentage of reported biological activity of *Aegle marmelos* linked with each compound, from all investigated articles.

Pharmacological activity is essential in herbal plants. The various acts of *A. marmelos*, which have been reported scientifically and investigated, have been illustrated in Figure 2.

### Anticancer activity

The *A. marmelos* of methanol and acetone extract of cytotoxicity against HEp-2, MDA-MB-231, and Vero cells were investigated. The IC_50_ for the methanol extract of *A. marmelos* was 47.08 g/ml, whereas the IC_50_ for the acetone extract of *A. marmelos* was 79.62 g/ml, making HEp-2 cells more sensitive to it. Both extracts of *A. marmelos* are toxic to cancer cells; however, Vero cells can survive 24 hours [47].

MTT assays on the human breast cancer cell line MCF-7 at various concentrations confirmed the *in vitro* anticancer activity. The flavonoids in fruit extracts act as a potential reducing agent and are reasonable for forming gold nanoparticles [48].

The aqueous fruit pulp extract from *A. marmelos* caused the most excellent MCF7 cell death at 100 g/ml and the _IC50_ at 47.92 g/ml concentrations [49].

In an *in-vivo* study, Swiss albino mice with Ehrlich ascites carcinoma received an intraperitoneal injection of a 400 mg/kg hydroalcoholic extract of *A. marmelos*. That significantly increased median survival time up to 28 days after tumor inoculation compared with the saline-injected control group [21]. The *A. marmelos* fruit pulp's ethanolic extract has anti-proliferative effect by inhibiting the proliferation of breast cancers in a rat model. Both the breast tumour volume (p < 0.05) and the different blood biomarkers (p < 0.0001) significantly decreased after *A. marmelos* treatment [50].

### Antidiabetic activity

The aqueous extract of *A. marmelos* fruits lowers blood sugar in streptozotocin-induced diabetes rat model. It boosts insulin secretion by partial regeneration from the β-cells of pancreatic islets [51]. The effects seen in the fruit extract-treated mice were better when compared with animals treated with glibenclamide. The present study's *in-vitro* assay demonstrated a potent antidiabetic effect from lectin extract, as measured by glucose uptake in yeast cells [52]. A fruit lectin extract with an IC_50_ of 3.36 μg/ml had greater efficiency than the usual medication metformin at increasing glucose uptake by yeast cells. This study found that *A. marmelos* fruit extract had hypoglycemic activity, which could be attributed to its antioxidant activity and high content of active constituents [53]. As a result, the various parts of *A. marmelos* plant could be beneficial as a portion of healthy food and in developing antidiabetic drugs. The active components in the leaf and callus materials reduce blood sugar levels in STZ-diabetic rabbits, and *A. marmelos* callus powder methanol extract is as powerful as the leaf extract in treating diabetes, as discussed in Table 5 [54]. This study indicates the aqueous seed extract of *A. marmelos* reduces the blood glucose level in normal as well as in severely diabetic rats and improves glucose tolerance in sub and mild diabetic animals and is referred to standard as tolbutamide [26]. The alcoholic extract of *A. marmelos* leaves significantly inhibited the enzymes α-amylase and α-glycosidase with IC_50_ values of 46.21 and 42.07 μg/ml, respectively. *A. marmelos* significantly reduced ROS levels that were elevated due to high glucose and enhanced glucose consumption in HepG2 cells (p < 0.05) [9].

**Table 5.** Antidiabetic activities of *Aegle marmelos*.

Part	Extract	Animal used	Standard	Inference
Fruit	Aqueous	Mice	Glibenclamide	lowers blood sugar and boosts insulin secretion
Fruit	Lectin	Glucose uptake by yeast cells	Metformin	IC_50_ of 3.36 μg/ml had greater efficiency than the usual medication
Various parts	Petroleum ether, methanol, chloroform, Benzene, aqueous	Streptozotocin diabetic Rabbit	–	Methanol extract showed maximum antidiabetic effect
Seed	Aqueous	Albino Wistar rats	Tolbutamide	reduces the blood glucose level
Leaves	Ethanol	α-amylase and α-glycosidase HepG2 cells	Acarbose	α-amylase and α-glucosidase were found to be IC_50_ 123.65 μg/ml and IC_50_ 141.56 μg/ml. Reduced ROS levels and enhanced glucose consumption (p < 0.05).
Leaves	Chloroform, butanol, and water	Streptozotocin induced Male albino rats	Metformin	Lowering the blood glucose levels

### Anti-inflammatory & antipyretic activity

The study examined the potential anti-inflammatory activities of the repeated extracts from *A. marmelos* leaves. An apparent analgesic effect was demonstrated in mouse models of carrageenan-induced paw edema and cotton-pellet granuloma to establish the antipyretic and analgesic activities of the leaf extracts. Additionally, the early and late phases of paw licking were diminished, and hyperpyrexia decreased [55]. In another study, the anti-inflammatory properties of the aqueous extract of *A. marmelos* dried flowers are investigated in Wistar rats. The anti-inflammatory effects of water extract were most effective at 200 mg/kg two hours after administration [56]. Aqueous extract from unripe *A. marmelos* fruit was found to have a dose-dependent impact in a different investigation focused on inflammatory bowel disease in albino Wistar rats. With much higher SOD and lower MDA levels and defense against mast cell degranulation, *A. marmelos* fruit had anti-inflammatory, antioxidant, and mast cell stabilizing properties [57].

### Antimalarial activity

*In vitro* antimalarial activity of *A. marmelos* leaf methanol extract, which showed the highest activity against *Plasmodium falciparum*, elicited low cytotoxicity, and the promising antiplasmodial activity of *A. marmelos* of IC50 is found to be 7 g/ml [58]. Infected mice with a suppressive effect on the parasite did not respond to *C. longa* treatment; however, *A. marmelos* at 20 and 40 mg/kg body weight inhibited parasite infection. Finally, *A. marmelos*, demonstrated strong antioxidant and antiplasmodial properties; it could be one of the traditional plants used to treat malaria [59]. With an IC_50_ of 500.06 ppm, standard *Temephos* has better larvicidal activity toward *Anopheles stephensi* when compared with crude leaf extracts of *A. marmelos* Correa [60].

### Antimicrobial activity

The antimicrobial activity of *A. marmelos* is discussed briefly in Table 6 respectively. *Candida albicans, Aspergillus niger, Aspergillus fumigatus*, and *Staphylococcus aureus* all had MIC (Minimum inhibitory concentrations) values of 19.5 g/ml, 39 g/ml, 625 g/ml, and 1.25 g/ml, respectively [61]. When used against *Candida albicans* and *Aspergillus niger*, it showed practical MFC (Minimum fungicidal Concentration) values of 2.5 mg ml^-1^ and 5 mg ml^-1^, respectively. In the present review, the decoction was more effective against fungi than food-pathogen bacteria. The control drug ampicillin was identified to be effective as similar to the ethanolic extract of *A. marmelos* fruit pulp by inhibiting the growth of pathogenic bacterial strains [62]. The antibacterial activity of the different *A. marmelos* leaf extracts was tested using the disc diffusion method on multi-resistant strains of bacteria. From there, it can be shown that the pet ether extract exhibits greater action than regular streptomycin [63]. In the ethyl acetate extract of *A. marmelos* leaf, the quinine compound was identified and possessed good antibacterial activity against gram-positive and negative bacteria [64].

**Table 6.** Antimicrobial activity of *Aegle marmelos*.

Plant part	Extract	Method	Organism	Standard
Leaves	Ethyl acetate	Disc-diffusion method	*E. coli*, *S. typhii*, and *P. aeroginosa*	Streptomycin
Fruit pulp	Aqueous, Ethanolic, and petroleum	Standard tube dilution technique	*Staphylococcus aureus*	Ampicillin
Leaves	Pet ether	Disc-diffusion method	Multi-resistant strains of bacteria	Streptomycin

### Antioxidant activity

Antioxidants are organic complexes that can safely interplay with free radicals and stop the chain reaction before harming fundamental molecules. Free radicals are highly reactive molecular species containing one or more unpaired electrons. They are generated from regular metabolism while using O_2_ to burn food for energy [65]. It is generally known that reactive oxygen species (ROS) play a role in developing several illnesses, including cancer and cardiovascular disease. Plants include antioxidants or polyphenols that can successfully neutralize these ROS and prevent the spread of disease [66]. Oxidative stress is produced during normal metabolic processes in the body and induced by various environmental and chemical factors, which causes the generation of various reactive free radicals and subsequent damage to macromolecules like DNA, proteins, and lipids. In comparison to standard - gallic acid (IC_50_ 1.1 ± 0.08 μM), marmelosin exhibited potent antioxidant activity with an IC_50_ of ∼15.4 ± 0.32 μM in ethyl acetate extract of bael fruit. Marmelosin was discovered to have better antioxidant properties than standard gallic acid [67]. In this investigation, the *A. marmelos* fruit decoction showed good antioxidant activity with an IC_50_ of 17.37 ± 2.71 mg/ml and 379.9 ± 28.28 mg AEAC/100 g for standard ascorbic acid [61].

### Antispermatogenic activity

In *A. marmelos* bark extract, marmin and fagarine are high, reducing male fertility [68]. The ethanolic extract of *A. marmelos* bark on sperm motility was reported to have a beneficial effect on sperm locomotor activity. It has also been reported that increasing the concentration of extracts reduces sperm motility. The alkaloids isolated from *A. marmelos* leaf were significantly decreased the fertility in male albino rats in dose dependent manner [69]. *A. marmelos* extract is an excellent choice for male contraception, the extract has the ability to completely suppress pregnancy and restore fertility rapidly after treatment cessation [68]. The male albino rats reproductive systems were subjected to three various doses of a 50% ethanolic extract from *A. marmelos* leaves: 100, 200, and 300 mg\kg 1 day 1 for each rat for 60 days. All of the significant accessory sex organs shrunk after ingesting the extract [70]. The cauda epididymis of the treated animals produced considerably less sperm, both in terms of motility and density. Male rat fecundity was completely decreased by *A. marmelos* at 300 mg.

### Antiulcer activity

Methanolic and aqueous extracts of *A. marmelos* seeds were tested for antiulcer activity in indomethacin-induced ulceration, stress-induced ulceration, and pylorus ligation-induced ulceration by using ranitidine as standard (50 mg/kg) [71]. Peptic ulcers are caused by the bacteria *H. pylori*. There is little or no literature on the effect of *A. marmelos* on Helicobacter pylori, so more research is required to determine its effect on H. pylori. If it positively reduces AMR, it will be an excellent herbal drug to treat abscesses with no adverse effects [72]. *A. marmelos* is frequently used to heal ulcers and related illnesses in Ayurveda and observed for the oral administration of methanolic extract of *A. marmelos* for affected rats with stomach ulcers induced by lipopolysaccharide caused by Helicobacter pylori [73]. A dose of 500 mg/kg of methanolic extract was shown in the trial to reduce stomach ulcers by 93.98%. Gastric secretory parameters, such as free and total acidity, acid output, stomach juice volume, and pepsin concentration, were inhibited, resulting in decreased gastric ulcers.

### Antiviral activity

Different portions of the *A. marmelos* are observed against human coxsackie viruses B1-B6 for *in-vitro* antiviral activity with ribavirin as a standard antiviral drug. Thus Marmelide possessed 32-times more potent inhibitory activity than ribavirin [74]. *A. marmelos* extracts were shown to be effective against the white spot syndrome virus in shrimp at a dose of 150 mg/kg of animal body weight [75]. The isolated volatile oil from *A. marmelos* is examined for its ability to inhibit the growth of eight different types of fungi. At 0.05% concentration, the essential oil completely prevented all fungi from producing spores. The majority of the fungus is significantly inhibited at around 75% and 90% at 0.03% and 0.04%, respectively. At concentrations of 0.03% and 0.04% of the oil, the most resistant strain, *F.udum*, showed 65% and 80% inhibition rates, respectively [76].

### Toxicity studies

*A. marmelos* dried fruit pulp is examined for its topical characteristics. Swiss albino mice were tested for acute oral toxicity with an ethanol extract of the dried fruit pulp from *A. marmelos* at 550 and 1250 mg/kg. Test results should indicate that the extract is not hazardous at these doses. Mice's behavior and physiological activity remained unchanged (14 days) throughout the trial [43]. The findings showed that the test extract's LD_50_ is highly significant. The oral acute toxicity study did not show any toxic symptoms, changes in behavior, or mortality at 1250 mg/kg doses. Thus, the ethanolic extract of *A. marmelos* dried fruit pulp extract has no discernable biologically significant toxic effect on the mice below LD_50_.

## Discussion

The biological actions of isolated compounds from *A. marmelos* that are being investigated using extracts can be connected to this review. This investigation concludes that *A. marmelos* has a promising future in treating and preventing different ailments, including cancer, infectious disorders and diabetic conditions. Reviews on spermatogenic, analgesic and antipyretic, inflammatory, antiulcer, and malaria treatment drugs are only a few topics covered in these reviews. For this reason, it is essential to develop clinical research on this medicinal plant and learn from traditional healers who have gathered knowledge through many generations of trial and error. The use of bael has gained popularity worldwide as its beneficial characteristics are being researched to develop new treatments potentially. As a result, the demand for novel therapeutic drugs with focused action and limited adverse effects justifies further clinical and preclinical research on *A. marmelos*.

## Conclusion

These investigations have shown that *A. marmelos* has therapeutic potential and contains elements that could be used to make new medications for the prevention, mitigation, or treatment of diabetes, cancer, and a variety of pathogenic illnesses. *A. marmelos* has been historically used for a variety of ethno botanical purposes. Unfortunately, most compounds still need to be thoroughly assessed to investigate novel lead molecules or pharmacophores. Furthermore, the mechanisms of a few bioactive chemicals have been discovered so far. Comprehensive research is necessary to ascertain the mechanisms of action, the bioactivity of numerous phytochemicals, and the effectiveness of *A. marmelos* medicinal characteristics.

## Future perspective

This study concludes the various parts of *A. marmelos*; preclinical studies are performed for different activities. Many chemical compounds are isolated, but fewer studies are conducted. In the future, clinical trials will be conducted for those activities. The demand for Bael fruit is likely to increase due to its growing popularity as a health food and ingredient in various food and beverage products. Additionally, the growing interest in traditional and natural remedies for various health conditions is likely to drive demand for Bael fruit.

**Executive summary**

**Rutaceae**

- *Aegle marmelos* (Indian bael or bael fruit), Rutaceae family, tree species native to India and Southeast Asia.

***Aegle marmelos* Linn**

- Traditionally used for the treatment of various ailments, including diarrhoea, dysentery, and fever.

**Reported phytochemical & its activity**

- Phytoconstituents, including alkaloids, coumarins, tannins, flavonoids, terpenoids, saponins, and glycosides.

**Pharmacological activity**

- Antibacterial, antifungal, antiviral, antimalarial, and antiparasitic activities.
